# Reconstructing the historical decline of lichen cover across the reindeer fence of the Finnish–Norwegian border

**DOI:** 10.1007/s13280-025-02171-3

**Published:** 2025-05-13

**Authors:** Tuomo Wallenius, Jarle W. Bjerke, Rasmus Erlandsson, Tiina H. M. Kolari, Aleksi Räsänen, Teemu Tahvanainen, Hans Tømmervik, Emelie Winquist, Tarmo Virtanen

**Affiliations:** 1 University of Helsinki, Biocenter 3, Viikinkaari 1, P.O. Box 65, 00014 Helsinki, Finland; 2 Department of Arctic Ecology, FRAM –High North Research Centre for Climate and the Environment, Norwegian Institute for Nature Research (NINA), P.O. Box 6606, 9296 Langnes, Tromsø, Norway; 3 Department of Ecology, Environment and Plant Sciences, Stockholm University, 106 91 Stockholm, Sweden; 4 Centre de recherche sur la dynamique du système Terre (GEOTOP), Université du Québec à Montréal, C.P. 8888, Succ. Centre-Ville, Montréal, QC H3C 3P8 Canada; 5 Department of Environmental and Biological Sciences, University of Eastern Finland, Yliopistokatu 7, P.O. Box 111, 80101 Joensuu, Finland; 6 Geography Research Unit, University of Oulu, Pentti Kaiteran Katu 1, PL 8000, 90014 Linnanmaa, Finland; 7 University Centre in Svalbard, P.O. Box 156, 9171 Longyearbyen, Svalbard Norway

**Keywords:** Grazing, Herding, Lichen, Reindeer, Remote sensing

## Abstract

We analysed the history behind the current contrasting lichen covers of two adjacent reindeer herding districts at the Finnish–Norwegian border. We conducted vegetation field inventories across the border fence and reconstructed a lichen cover history from 1959 to 2020 using aerial and satellite images. The oldest images showed only a slight difference in lichen cover between the different sides of the border fence. Since the late 1950s, lichen cover has decreased in both districts. At present, lichen biomass is approximately three times greater in in the Norwegian winter pasture than in the Finnish herding district, which has less strictly defined seasonal pastures. A lichen biomass model indicated that lichen intake by reindeer cannot explain the decline in lichen biomass in either of the districts. We suggest that the lichen decline is mainly due to trampling and foraging-induced loss, while other unknown ecological and climatological factors may also be involved.

**Supplementary Information:**

The online version contains supplementary material available at https://doi.org/10.1007/s13280-025-02171-3.

## Introduction

Large grazers and browsers have an immense impact on the global environment, shaping the structure and diversity of many ecosystems. These effects became increasingly governed by humans with the invention of animal husbandry, and currently, livestock biomass outweighs wild animal biomass multiple times (Greenspoon et al. 2023). While much attention has been given to productive tropical and temperate habitats and intense land use, animal husbandry at relatively low population densities can affect landscapes in less-productive environments, such as Arctic tundra and subarctic woodlands (Mysterud 2006; Stark et al. 2023).

*Rangifer tarandus*, known as reindeer in Eurasia and as caribou in North America, is a circumpolar ungulate species with high ecological, cultural, and economic value. The reindeer is considered a semi-domesticated species, as wild populations exist in northern Eurasia in addition to the herded populations. In many regions of the circumpolar tundra, the reindeer is the only large herbivore affecting vegetation (Bernes et al. 2015), and most populations migrate annually over hundreds of kilometres between summer and winter pastures.

Reindeer demonstrate seasonal dietary shifts, with summer diets mainly containing forbs, graminoids, and deciduous shrubs, shifting to primarily mushrooms in autumn and lichens in winter (Kojola et al. 1995; Webber et al. 2022). The importance of ground lichens in the reindeer diet is manifested in many languages in the names of certain widespread species belonging to the *Cladonia* genus. For example, *Cladonia rangiferina*—“ranesjeagil” in North-Sámi, the Finnish name “palleroporonjäkälä” for *Cladonia stellaris,* and the Norwegian name “lys reinlav” for *Cladonia arbuscula* all refer to reindeer in their names, as they are preferred winter forage of the species.

The wild reindeer has been the most important subsistence animal for people of northern Fennoscandia for thousands of years (Gjerde 2019; Harlin et al. 2019). Domestication of reindeer started around 1300 CE (Salmi et al. 2021) or possibly earlier in the first millennium CE (Bjørklund 2013). In northern Scandinavia, reindeer husbandry with large semi-domestic reindeer herds was developed in the late Middle Ages (Røed et al. 2018). During this time, the annual migrations with animals continued, but an increasing proportion of the reindeer were owned and herded by nomadic Sámi people. During the twentieth century, traditional reindeer husbandry transformed into a modern livelihood with ecological, cultural, and economic implications (Helle and Jaakkola 2008). Today, there are more than 600 000 semi-domesticated reindeer in Fennoscandia (Bernes et al. 2015), and reindeer husbandry is regulated by national laws and decrees.

A strong reindeer grazing pressure has long been known to lead to a reduction in ground lichen biomass (Komiteanmietintö 1905; Turi 1910; Stark et al. 2021), which in turn has been considered the limiting factor for reindeer numbers (Mattila 1981; Bernes et al. 2015). The etymology of the word ‘grazing’ refers to feeding on grass, and therefore, the common association is that reindeer have reduced the lichen cover by feeding on it. However, consumption is not the only way reindeer affect the lichen cover. Trampling during snow-free periods compresses the fragile thallus of lichen, which, in dry conditions, may have strong negative impact on the lichen cover (Heggenes et al. 2017). Snow cover protects lichen against trampling, but consumption alone does not explain all lichen loss in winter pastures (Gaare and Skogland 1980). Yet the level of reindeer-induced lichen loss due to trampling and during grazing has rarely been estimated, despite being an important ecological variable and a required input in ecological and economical reindeer pasture models (Pekkarinen et al. 2017).

From the late 1980s onwards, satellite image analyses indicated that northernmost Finland had a lower ground lichen cover compared to adjacent regions in Norway and Russia (Johansen and Tømmervik 1990; Käyhkö and Pellikka 1994; Väre et al. 1996; Kumpula 2006). The comparably thick lichen carpets in the Russian Murmansk region have been considered as a consequence of a low reindeer population; however, the reindeer population density in northern Norway was high and comparable to that in Finland (Väre et al. 1996). This indicated that the differences in lichen abundance between Norway and Finland could not be explained by reindeer population density alone.

The apparent decisive factor is the management practices that differ between the Fennoscandian countries due to historical geopolitical reasons. During the Finnish War in 1808–1809, the Russian Empire annexed the area of present-day Finland from the Kingdom of Sweden, and a new country border was drawn across ancient migration routes. From 1852, Finnish and Norwegian reindeer were no longer allowed to cross the country border (Komiteanmietintö 1905), and nomadic Sámi families had to choose whether to live with their reindeer in Finland, Sweden, or Norway (Lantto 2010). This did not stop the annual migrations but forced the pasture rotation to take place in considerably smaller areas, especially in Finland. The average size of herding districts in Finland is currently less than 2300 km^2^, whereas the herding range—prior to the border closure—was manyfold, extending across northern Sweden, Finland, and Norway.

Reindeer herding gradually adapted to the restricted area, delineating new pastures for summer and winter. However, breaches of the border closure act remained relatively frequent (Komiteanmietintö 1905; Anonymous 1954). In 1950, Finland and Norway began building a joint border fence that was completed in 1957 (Sara 1999). Fencing was an important cause of the transition from nomadic intensive herding to modern practices (Näkkäläjärvi 2007; Helle and Jaakkola 2008; Lehtola 2012). After the border closure, the small herding districts, along with the lifestyle changes and other land-use pressures, made it challenging to uphold separate winter and summer pastures on the Finnish side (Näkkäläjärvi 2007). The situation in Norway has been different; the reindeer populations continued their ancient migrations between summer pastures along the northern seacoast and winter pastures in the interior parts, close to the Finnish border (Riseth et al. 2016).

**Fig. 1:**
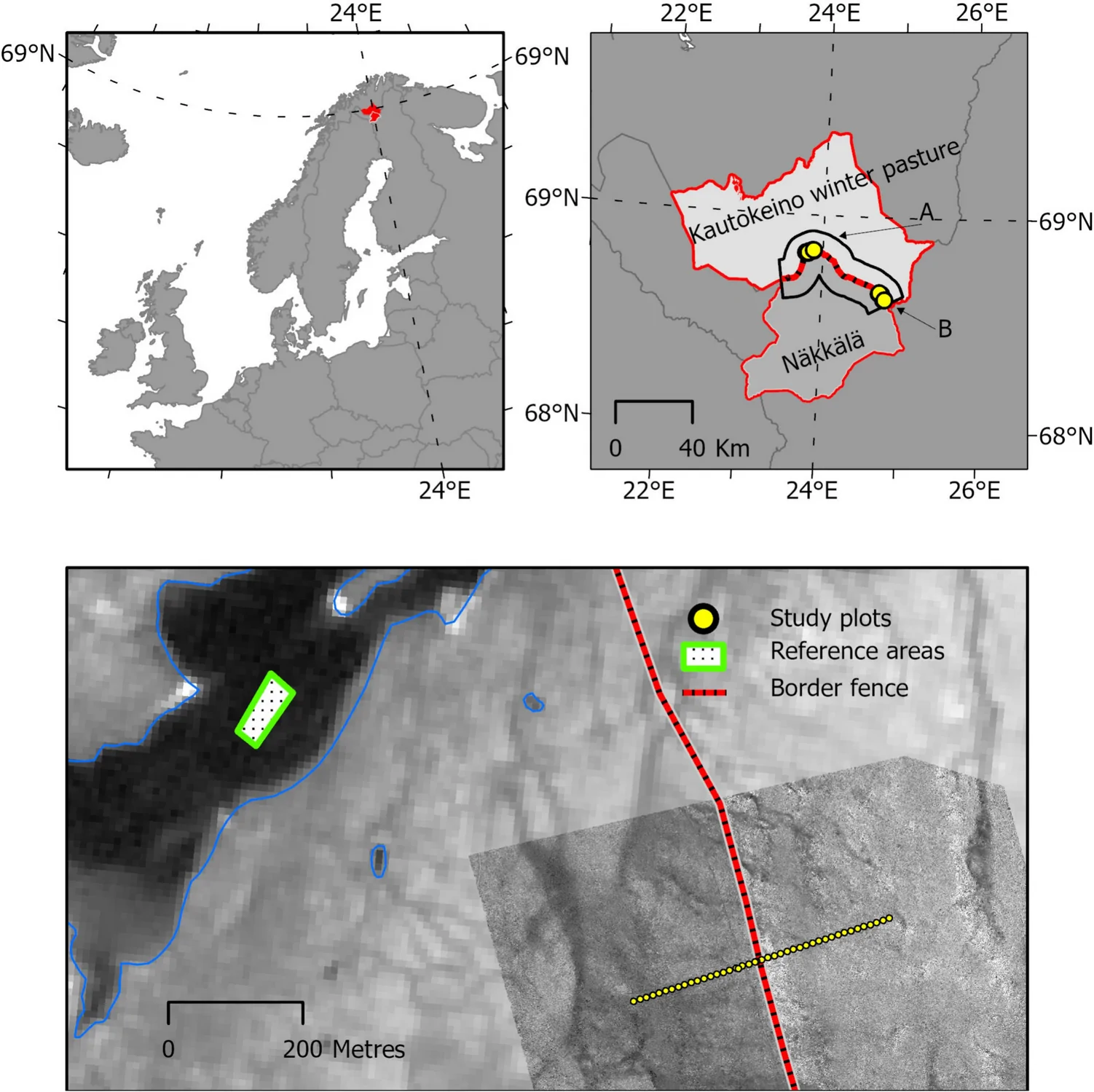
The study area comprises the Kautokeino winter pasture in Norway and the Näkkälä herding district in Finland. Letter A in the upper right figure refers to the Jauristunturit–Máðároaivi site and letter B to the Palokorsa–Sieiddečearru site. The lower figure shows one reference area on a lake on the Sentinel-2 image used for calibration and the study plots of one transect on a drone image mosaic in the Palokorsa–Sieiddečearru landscape

**Fig. 2:**
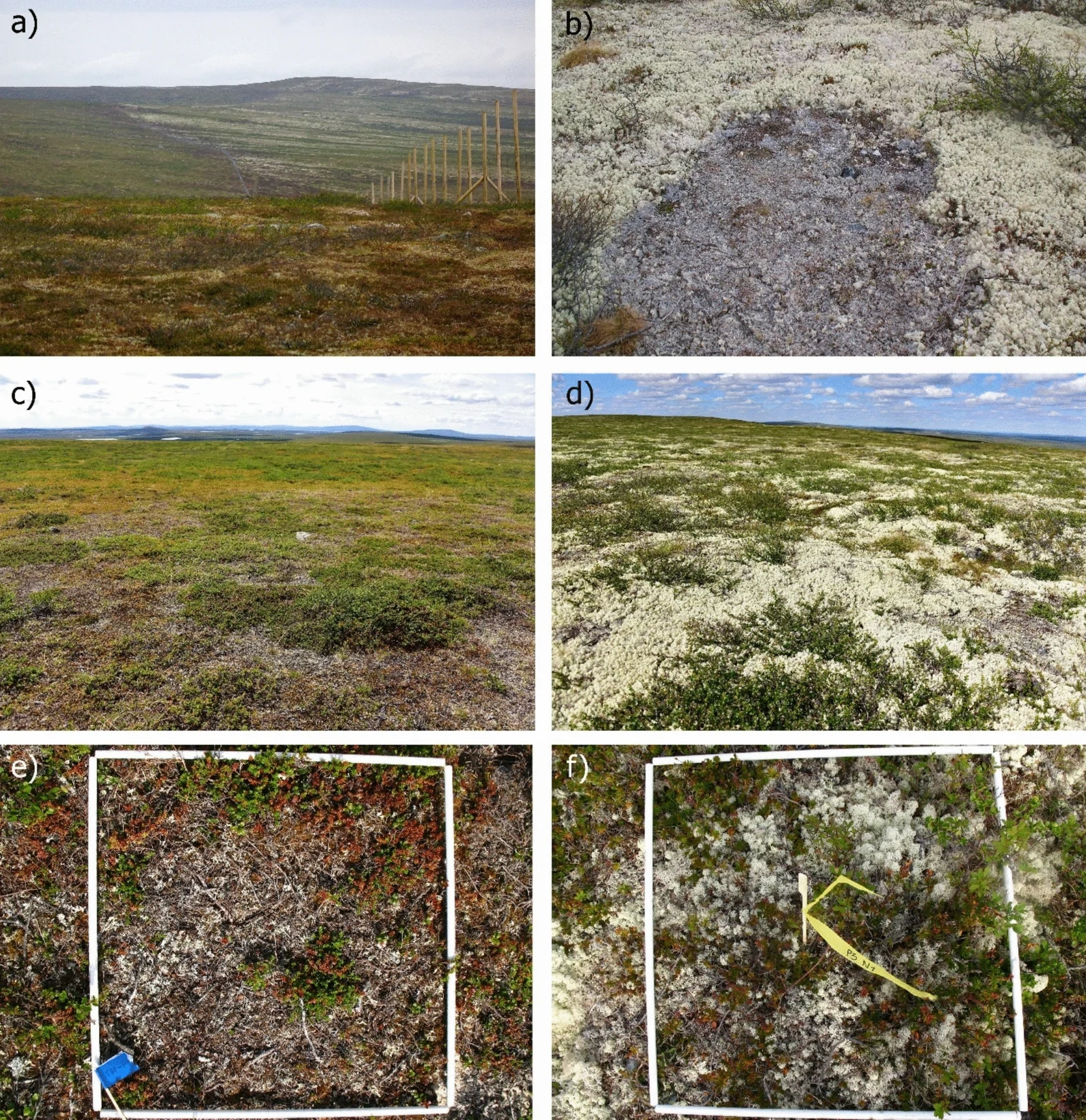
**a** Reindeer lichen pastures and the border fence. **b** Reindeer winter grazing pit on thick lichen carpets. **c** Pastures on the Finnish side. **d** Pasture with abundant lichen cover on the Norwegian side. **e** Study plot with a frame on the Finnish side and **f** in Norway. Photos by Tuomo Wallenius (a and b) and Tarmo Virtanen (c–f)

**Fig. 3:**
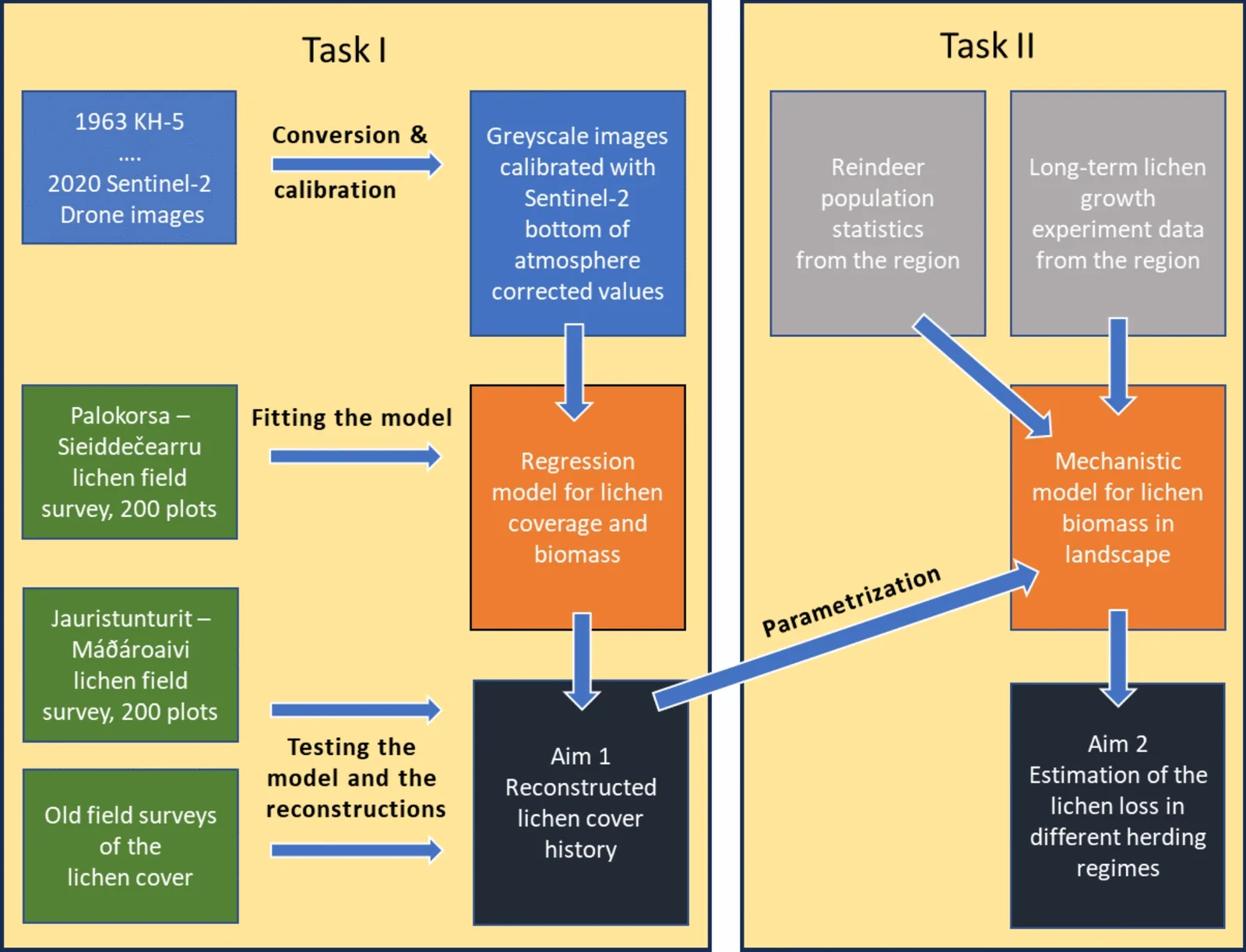
Workflow of the study project linking the data sources, tasks, and aims; blue: remote sensing imagery, grey: reindeer and lichen data from the same region, green: botanical field surveys, orange: models, black: results

We analysed the changes in lichen cover in two contrasting reindeer grazing regimes caused by the erection of the fence along the Finnish–Norwegian border (Figs. 1, 2). We aimed to (1) reconstruct the lichen cover (proportional coverage and biomass) history in the different pasture regimes from the late 1950s to 2020. To give insights on the causes of the lichen biomass changes, we also aimed to (2) estimate the magnitude of trampling and other loss of lichen biomass by reindeer in different pasture regimes. We addressed these aims in two steps (Fig. 3). The first task (I) was to develop a remote sensing method for lichen cover estimation utilizing historical greyscale aerial photographs and satellite images. The second task (II) was to parametrize a mechanistic model for reindeer lichen biomass in the landscape using satellite image-based lichen biomass estimates.

The remote sensing task builds on the fact that lichens have high reflectance values, and thus, they can be separated from mosses and other types of vegetation (Petzold and Goward 1988; Solheim et al. 2000). The most important terricolous reindeer forage lichens, such as the *Cladonia* species, are nearly white, pale grey, or yellowish. The same applies to the genera *Flavocetraria* and *Stereocaulon*. In this study, we focus on these three genera and refer to them hereafter as forage lichens, as they constitute the majority of reindeer winter forage.

## Materials and methods

### Study area and the conditions for reindeer pastoralism

The study area comprises two contiguous areas: (a) the entire Näkkälä reindeer herding district (3557 km^2^) in Enontekiö, Finland, and (b) the West Finnmark winter pasture area (5808 km^2^) in Kautokeino, Norway (Fig. 1). These areas are separated by a reindeer fence that runs parallel to the national border, albeit with some minor deviations (Fig. 2).

The whole study area predominantly lies on granitic rocks of the Fennoscandian Shield and experiences relatively continental climates (Oksanen and Virtanen 1995). The topography of the area is characterized by undulating terrain, with a mean elevation above sea level of 377 m on the Finnish side and 424 m on the Norwegian side (Global Multi-resolution Terrain Elevation Data 2010 courtesy of the U.S. Geological Survey). Based on the Era5 land climate reanalysis (Muñoz Sabater 2019), the average annual temperatures for the period 2012–2020 were − 0.5 °C on the Finnish side and − 0.8 °C on the Norwegian side of the study area. Correspondingly, the average annual precipitation was 684 mm on the Finnish side and 665 mm on the Norwegian side. Snow cover typically persists from October to late May.

The study area belongs to the northern boreal vegetation zone, with extensive zones of oro-arctic or alpine vegetation (Ahti et al. 1968; Oksanen and Virtanen 1995). On the Norwegian side, forests cover 41.1%, peat bogs 28.2%, tundra heaths and other sparsely vegetated areas 27%, and lakes and rivers 3.6% of the landscape (European Environment Agency 2020). The corresponding figures for the Finnish side of the study area are 41.9%, 31.7%, 21.5%, and 3.4%, respectively. A marked difference between the herding districts is that Scots pine (*Pinus sylvestris*) forms forests in the southern part of the Finnish side, whereas Norwegian forests are dominated by mountain birch (*Betula pubescens* ssp. *czerepanovii*). In northern Finland, pine forests provide lichen pastures with arboreal lichens on trees that are at least as suitable for reindeer as those found in tundra heaths and mountain birch forests (Kumpula et al. 2019).

Following the border closure, the Näkkälä region, which earlier primarily was used as a winter pasture, was divided into partly intermingled and overlapping winter, spring, summer, and autumn pastures (Kitti and Forbes 2006; Kitti et al. 2009; Stark et al. 2023). The Norwegian side of the study area has only been used as a winter pasture, with the summer pastures of Kautokeino district being located on the Finnmark and Troms coast, ca. 150–200 km north and west of the study area (Tømmervik et al. 2012; Stark et al. 2023). Considering that reindeer herding may have considerable effects on lichen pastures, background information on the changing conditions, realized grazing regimes, and the state of pastures from the 1500 s until modern times was collected from the literature (Table 1).

**Table 1:**
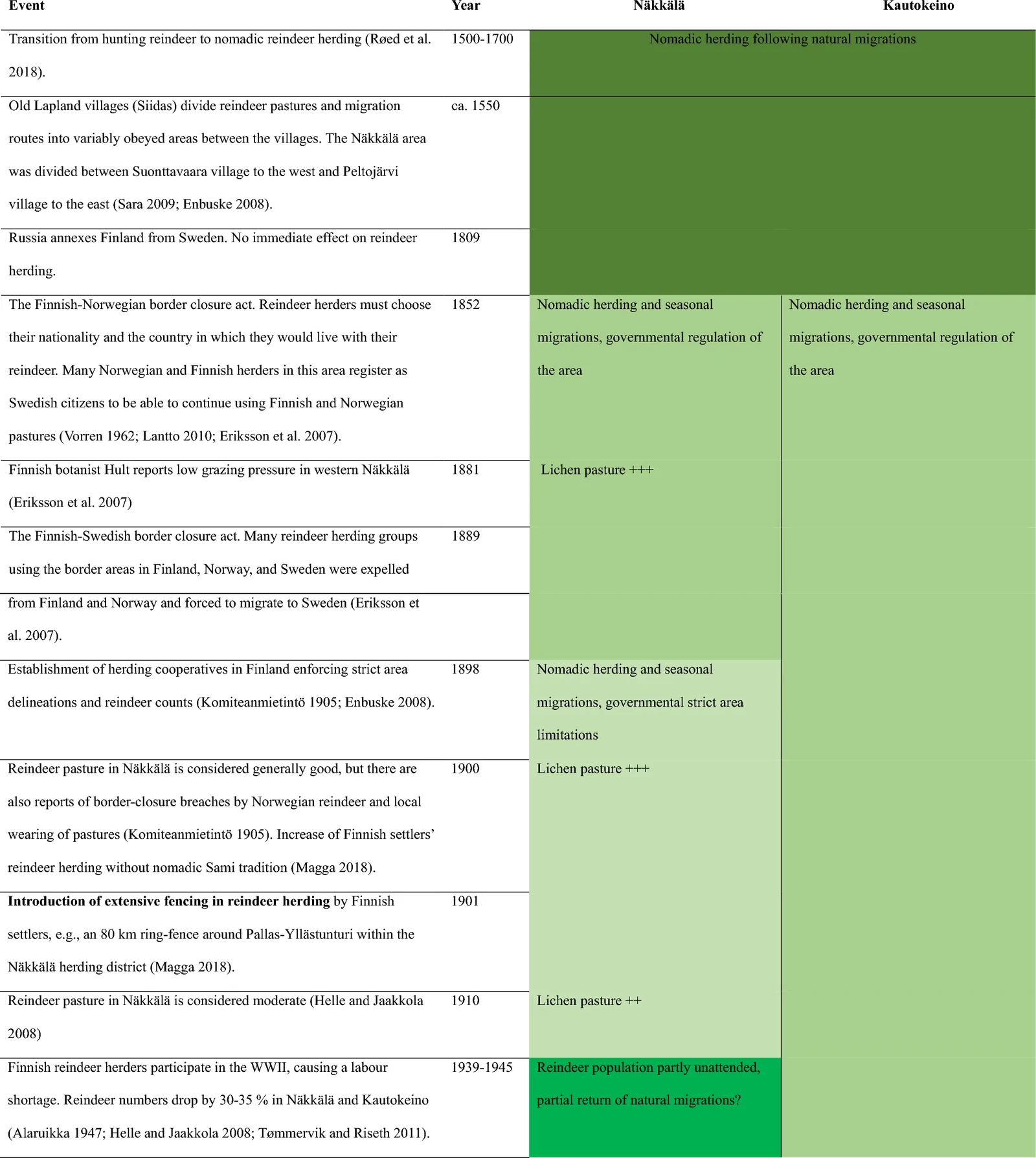

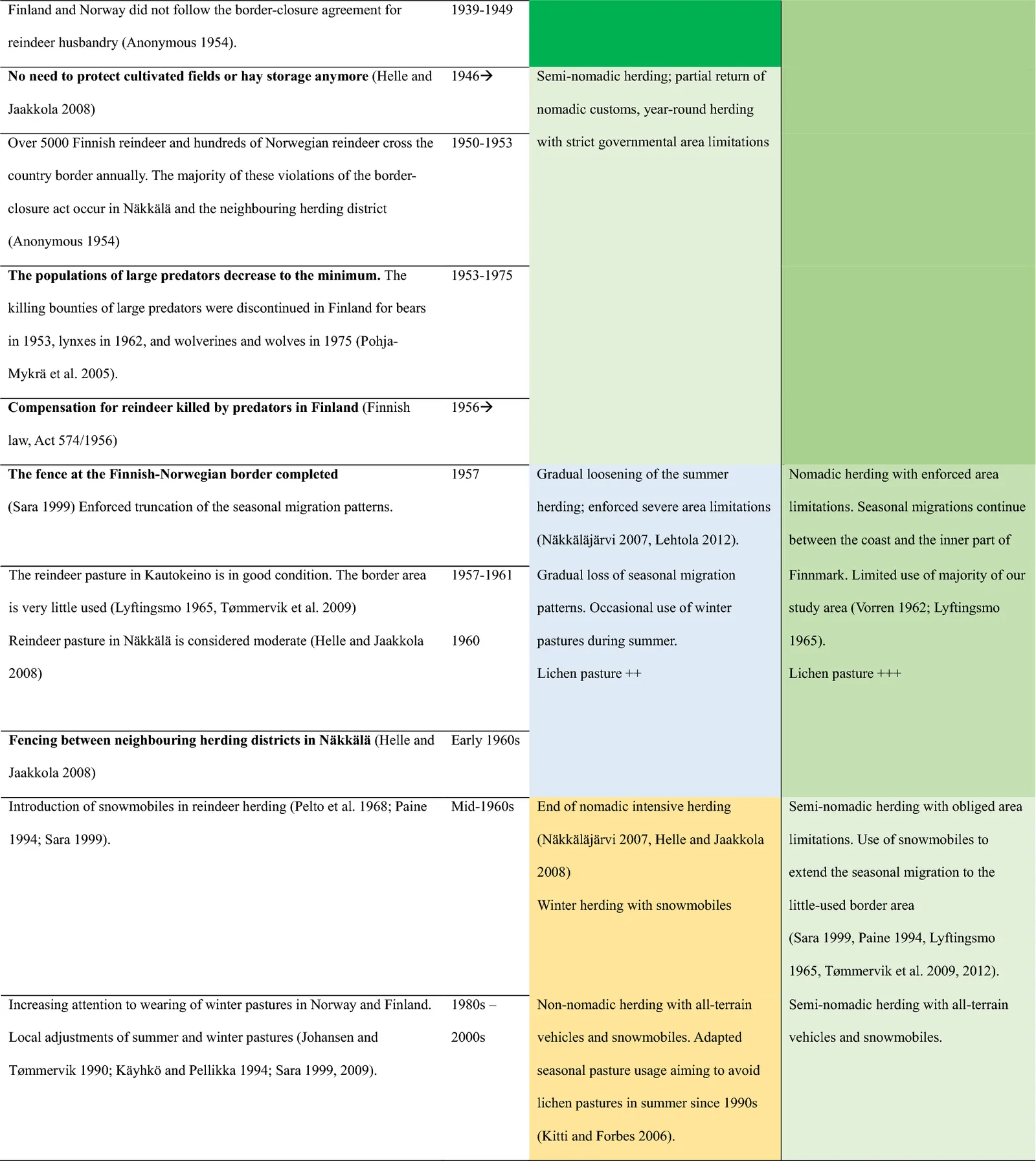
A timeline of changing conditions and realized reindeer grazing regimes in the two herding districts in our study area. The conditions written in bold are considered prerequisites for the change from intensive to extensive herding (see Helle and Jaakkola 2008). Early observations of the pasture conditions are marked as follows: +++ = good, ++ = moderate. Changing background colours in the right columns depict different grazing regimes during the history of reindeer husbandry

### Vegetation surveys

Botanical field surveys were carried out in two sub-sites along the Finnish–Norwegian border, to compare the lichen cover of the different grazing regimes. Fieldwork was conducted in the Jauristunturit–Máðároaivi area in July 2020 and in the Palokorsa–Sieiddečearru area in July 2021. These two sites, ca. 40 km apart, are both characterized by treeless tundra heathland (Figs. 1, 2). The data from the first field campaign served as a ground reference for remote sensing-based mapping of lichen cover, and the second data set was used for testing the produced lichen cover maps (Fig. 3). At both study sites, we established five 400-m transects that were located 0.5–2 km apart from each other and laid out perpendicularly across the fence (200 m on each side) in areas with similar topography and moisture conditions on both sides of the fence. Along the transects, we placed a total of 400 vegetation plots of 0.25 m^2^ (0.5 × 0.5 m) at 10-m intervals. The study plots were framed either with a string and nails in the corners of the plots or with a rigid plastic frame (Fig. 2e and f). We recorded the centre coordinates of every plot with a real-time kinematic Global Navigation Satellite System GPS, Trimble R10 or Topcon HiPer, with an accuracy of 2 cm. We visually estimated the coverages of all vascular plants, bryophyte, and lichen species in each plot. The approximate average lichen height was determined with a ruler at 1–4 subjectively selected points within each plot, depending on the abundance and location of individuals. In practice, lichen height denotes the mean thickness of the lichen mat.

The field-based measurements rendered three-dimensional (3D) cover estimates, meaning that they included the proportions of species beneath other species, resulting in total coverage sums exceeding 100% for most plots. These 3D cover estimates were used to calculate the forage lichen volumes in the study plots with the following formula:

$$v \, = \, ch,$$

where *v* is lichen volume in dm^−3^ m^−2^, *c* denotes lichen 3D coverage in percentages, and *h* is the mean lichen height in millimetres. Lichen biomass was estimated using the equation:

b=22v,

where *b* = lichen dry weight biomass g m^−2^, 22 is the weight of one dm^−3^ of lichen in grammes, and *v* = lichen volume in dm^−3^ m^−2^ (Gaare and Tømmervik 2000; Tømmervik et al. 2012). We chose this linear formula from the various equations depicting the relationship between lichen dimensions and biomass (e.g. Moen et al. 2009; Kumpula et al. 2014), as it has been used in Norwegian studies in the same region (e.g. Tømmervik et al. 2009, 2012).

Due to overlapping layers of species, parts of the 3D coverage that include overlapping layers and that reach values over 100% cannot be observed from the aerial photographs and satellite images. This is a problem of all remote sensing-based methods to estimate the lichen cover. To obtain the most accurate link between the lichen cover and reflectance values of greyscale images, we generated an objective estimate of the two-dimensional reindeer lichen coverage (2D cover summing up to 100% as the maximum) as follows: All plots were photographed from above with a digital camera, and a point intercept analysis of the photographs was performed with ArcGIS Pro using a grid of systematically distributed crosshairs that was overlaid on a photograph of each vegetation plot. Each crosshair was zoomed in, close enough to determine whether it was on a target forage lichen. The interpretation of 100 crosshairs was then linearly transformed into a 2D cover; for instance, 50 crosshair hits indicated 50% cover and 25 hits 25% cover. Due to missing plot photographs, the point intercept analysis was performed only on four transects in both landscapes, i.e., on 320 study plots in total.

### Reconstructing forage lichen cover

We reconstructed the history of the forage lichen cover using multitemporal and multiresolution remote sensing data, including old black-and-white aerial photographs and satellite images. The reconstruction method is based on the observation that forage lichens are generally brighter, i.e. they reflect more light than vascular plants, bryophytes, peat, litter, bare soil, and lake surfaces (Petzold and Goward 1988). Dead grass, certain rock types, and sand are as bright or even brighter than forage lichens. However, grass, rocks, and sand do not cover large extents within the landscapes under study.

Our first step was to acquire the highest-quality cloudless imagery taken in July or August as far back in time as possible (Table 2). Images from late July were preferred, as this is peak growing season. Sentinel-2 satellite images were downloaded from ONDA.^1^Keyhole and Landsat satellite images were acquired from Earth Explorer^2^. Atmospherically corrected level 2 surface reflectance products were selected from Landsat 5–8 and Sentinel-2. Landsat 1 and 2 images were level 1 georeferenced products lacking the atmospheric correction. All other imagery was unprocessed. Clouds and their shadows were removed from the selected images using quality analyses or manual delineation. The selected satellite images covered 67–100% of the analysed area (Table 2).

**Table 2:** Information on the used aerial and satellite images and their calibrations. The image coverages were calculated from the non-forested area that was under the study. The calibration equations describe the linear regression lines between the reference areas’ pixel values in the different source images and the 30 July 2020 Sentinel-2 target image. In the equations, x denotes black-and-white raster values of the image in question. Coefficients of determination are provided as *R*^2^. *The aerial images were used only for the 400-m-wide zone at the border fence

Platform	Dates	Image coverage (%)	Pixel size	Calibration equation	*R*^2^
Airplane	1959-07-21	25*	0.28 m	*y* = 13.602*x* − 502.88	0.90
Airplane	1961-09-06	75*	0.40 m	*y* = 15.188*x* − 684.27	0.88
Keyhole 5	1963-08-29	100	40 m	*y* = 16.137*x* − 475.51	0.91
Landsat 1	1973-07-23	100	60 m	*y* = 36.921*x* − 909.53	0.87
Landsat 2	1980-07-30	100	60 m	*y* = 47.036*x* − 1105.5	0.90
Landsat 5	1984-07-09	67	30 m	*y* = 0.3307*x* − 2595.8	0.99
Landsat 5	1992-08-23	95	30 m	*y* = 0.3937*x* − 3247.3	0.96
Landsat 5	1997-07-11	70	30 m	*y* = 0.3222*x* − 2462.2	0.99
Landsat 7	2000-07-29	78	30 m	*y* = 0.3279*x* − 2441.4	0.98
Landsat 5	2009-08-27	99	30 m	*y* = 0.382*x* − 2992.5	0.95
Landsat 8	2013-07-23	85	15 m	*y* = 0.5081*x* − 3263.3	0.99
Sentinel-2	2020-07-30, 2020-07-31	91	10 m	*y* = *x*	1

Aerial images from 1959, covering an approximately 20-km sequence of the border between Näkkälä and Kautokeino winter pasture, were acquired from the Norwegian Mapping Authority. Aerial images from 1961 were retrieved from the National Land Survey of Finland.

High-resolution imaging of the study sites, using a DJI Matrice 300 drone equipped with a MicaSense RedEdge-M™ multispectral sensor, was also conducted during the field work. The Agisoft Metashape Pro 2.0.4 software was used to produce accurately positioned image mosaics from the 1961 aerial images and from the drone images. These mosaics were georeferenced in ArcGIS Pro using the most recent aerial orthophotographs provided by the Finnish Land Survey. The 1959 images were georeferenced and mosaiced manually in ArcGIS Pro.

To harmonize all images, we converted multi-band images to greyscale (one band) in ArcGIS Pro using equal weights for red, green, and blue channels. The early Landsat (1–3) images did not have a blue channel, and therefore, only red and green channels were used with equal weights.

Ideally, all the images would be in the same spatial and spectral resolution, but this was not possible due to the multisource nature of remote sensing data, and because the botanical survey is not practical to carry out at the scale of the satellite image pixels. The drone image resolution corresponded with the plot scale vegetation mapping, and the drone imagery hence offered us a possibility to link a certain range of reflectance values and lichen cover characteristics, thereby linking the study plots with the landscape scale (see the next two sections and Figures S1, S2, S3, and S4). The recorded measurements of lichen cover and biomass in the study plots represented the entire range of values found in the landscape during the study period. To test the effect of varying pixel size on our estimates, we resampled and aggregated three high-accuracy images into coarser resolutions of up to 60-m pixel size, which was the largest pixel size in our source material. After computing the lichen biomass from the same image but different pixel sizes, it was possible to see the effect of changing resolution.

### Image calibration

To be able to reconstruct the lichen cover history since the 1950s, we had to utilize images from several different platforms using various sensors and cameras, which were not directly comparable. To make the greyscale values comparable, all images were calibrated with a bottom of atmosphere reflectance Sentinel-2 image from 30 July 2020. This cloud-free image was of excellent quality and taken just two weeks after the first field campaign.

Satellite and old aerial images were calibrated using 48 reference areas delineated from 24 lakes, 9 open fens, and 15 sandpits or sandy areas. The reference areas varied in shape and size. The average size of sandpits and fens was about 0.3 ha, whereas the average size of lakes was about 2.2 ha. A comparison of the topographic maps and old aerial images suggested that the sandy areas have not moved and—together with lakes and fens—can be considered to have remained essentially the same during the study period. Lakes and fens have dark surfaces, whereas sand pits and beaches appear as bright spots in the black-and-white images.

To calibrate an old image, we took the average greyscale values of each reference area and compared them to values obtained from the same reference areas in the greyscale Sentinel-2 image. First, linear regression equations between these value pairs were computed in Excel and then used in ArcGIS Pro to calibrate different image sources (Table 2, Fig. S1). The calibration enabled comparing the satellite images to the Sentinel-2 image from 30 July 2020 (Fig. S2).

For calibrating the drone images, we assumed that the relatively short temporal and seasonal deviations between the fieldwork periods (13–16 July 2020 and 24–27 July 2021 and Sentinel-2, 30 July 2020) were insignificant considering the slow vegetation changes in the study area. The drone images were calibrated in two steps: (1) a coarse calibration into the same scale of raster values using linear regression between the greyscale values from the same random points from the drone mosaic and the Sentinel-2 image, and (2) a local refinement using the difference between the Sentinel-2 image and step 1 results. Subtracting the difference from the step 1 result gave us a high-resolution image with similar colour balancing as in the Sentinel-2 image. The second step revealed and removed errors in the colour balancing of the drone images that were caused by changing light conditions during the drone flight and by the mosaicking software (Fig. S3).

#### Forage lichen cover and biomass mapping

**Fig. 4:**
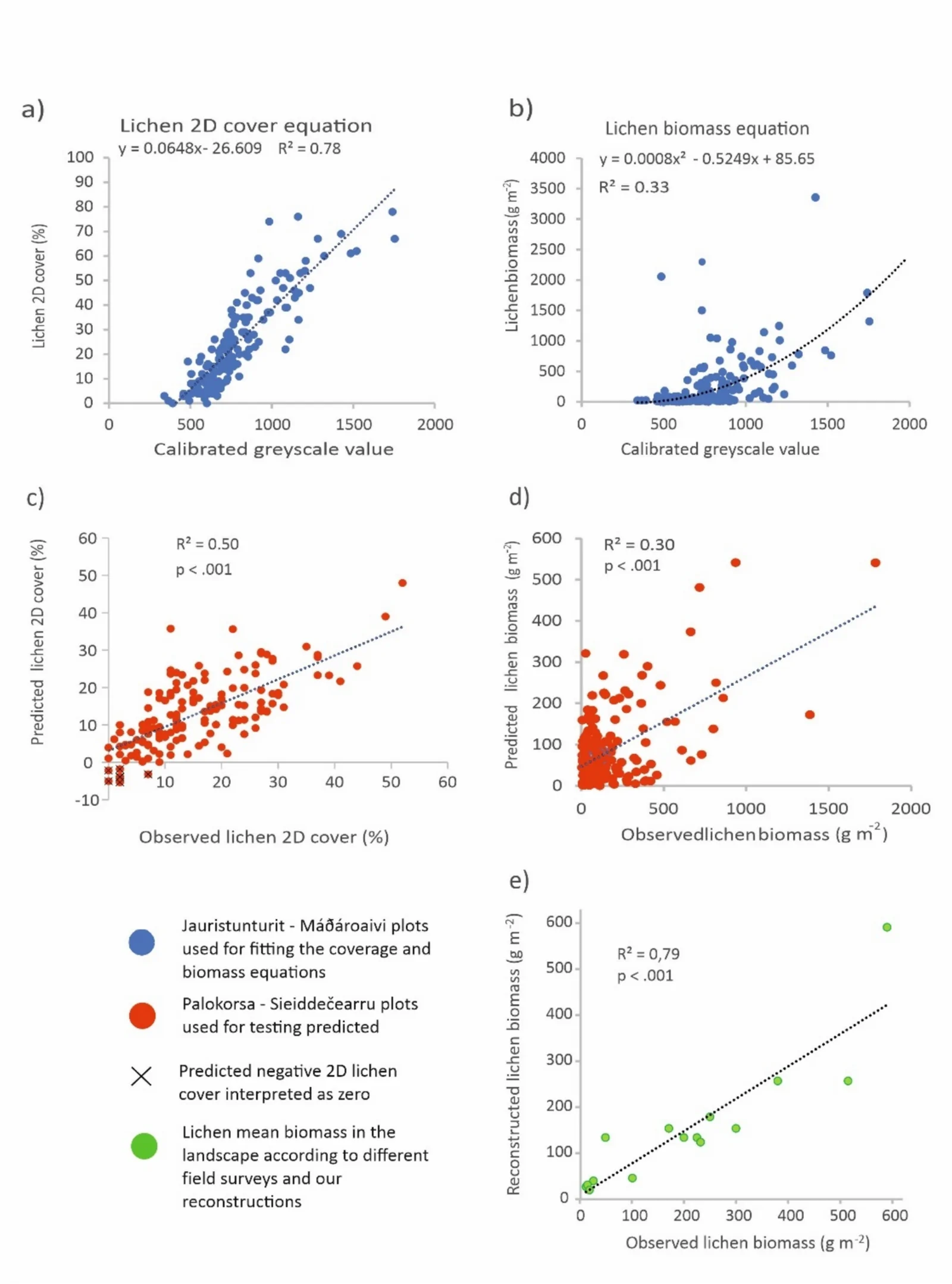
Lichen 2D cover (**a**) and biomass (**b**) equations fitted for the plots from the Jauristunturit–Máðároaivi area. These were used for predicting lichen 2D cover (**c**) and biomass (**d**) in the plots on the Palokorsa–Sieiddečearru site. Below the greyscale value 412, the lichen cover model predicts negative coverage. Negative coverages were reclassified as zero lichen coverage in the produced maps and analyses. Independent field surveys (Table S3) were utilized to test landscape-scale lichen biomass predictions (**e**)

**Fig. 5:**
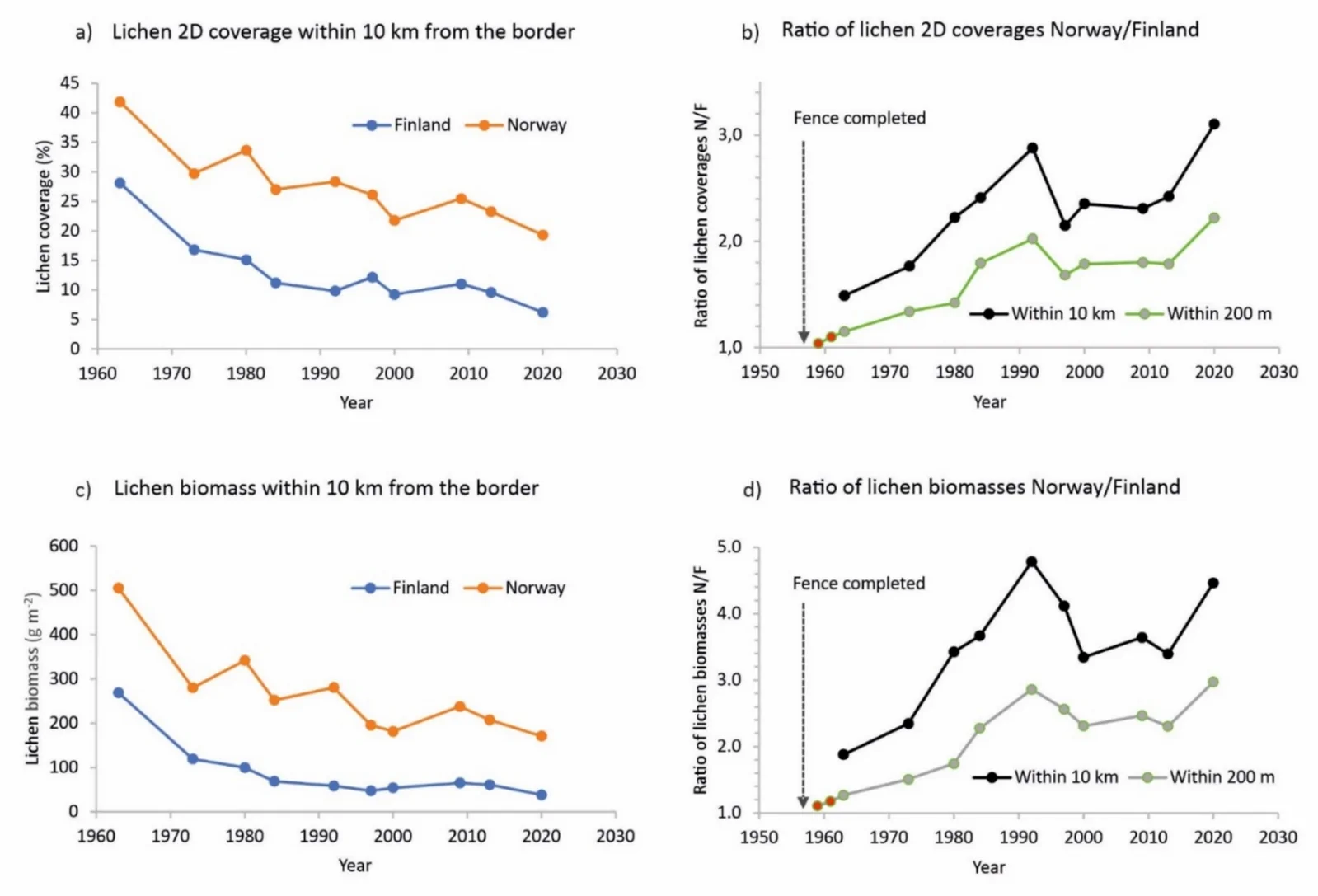
Comparison of lichen cover in different management areas. To render the comparisons unbiased, the areas of lakes, mires, and forests were excluded from the analysis. Reconstructed lichen 2D coverages, biomasses, and their ratios between Finland and Norway within 200 m and 10 km of the border. Red dots denote data points reconstructed from the aerial images

The drone image mosaics helped to bridge the gap between the size of the study plots (0.5 × 0.5 m) and the pixel size in Sentinel-2 (10 × 10 m). The average pixel values from a circular (*r* = 0.35 cm) area (0.4 m^2^) on the quadratic study plots were compared with the forage lichen 2D coverages (the point intercept analysis) and plot-level biomass data (Fig. S4). Regression equations were fitted with the plot data from the Jauristunturit–Máðároaivi study site and used for predicting forage lichen 2D coverage and biomass on the Palokorsa–Sieiddečearru plots (Fig. 4). The same equations were then used to estimate the lichen coverage and produce the biomass maps based on the various calibrated satellite images in ArcGIS Pro (Table 2, Fig. 5).

Due to the limited areas covered by images in Finland (1959) and Norway (1961) and the difficulties in compiling image mosaics with good colour balance, the old aerial photographs were not used for reconstructing lichen coverage and biomass for the whole study area. However, the images covered a large part of the border, extending at least 200 m on both sides; hence, they provided a relative measure of lichen cover in the early years after the fence was completed. A comparison of the lichen cover within 200 m and 10 kms of the border was performed in both countries. To make the areas as comparable as possible, we focused on tundra heaths. We excluded mires, lakes, sandy areas, and forests from the comparison using CORINE landcover classification with a 100-m resolution (European Environment Agency 2020). However, for modelling purposes we computed average lichen biomass for the entire herding districts of Näkkälä and Kautokeino winter pasture, including mires. Forested areas were masked out of the analysis because the method underestimates the lichen cover of sites shadowed by tree canopy.

#### Testing the lichen cover estimates

Lichen cover and biomass estimate accuracies were tested with the vegetation plot data from Palokorsa–Sieiddečearru. In addition, we tested how well our historical lichen biomass reconstructions fit the field surveys of lichen biomass in Kautokeino (see the compilation of Norwegian studies by Tømmervik et al. 2009 and 2012) and Näkkälä (Mattila 1981; Kumpula et al. 2014, 2019; Table S3). Each field survey was compared with the temporally nearest reconstruction from the same area (Table S3). The Norwegian lichen estimates did not include bogs; therefore, we compared lichen estimates to corresponding computations without bogs (Table 3). To estimate the error in the lichen biomass reconstructions, we computed—using the same data—a weighted mean absolute per cent error (wMAPE):

$$w{\mathrm{MAPE}} = \frac{{\mathop \sum \nolimits_{i = 1}^{n} |O_{i} - S_{i} |}}{{\mathop \sum \nolimits_{i = 1}^{n} |O_{i} |}},$$

**Table 3:**
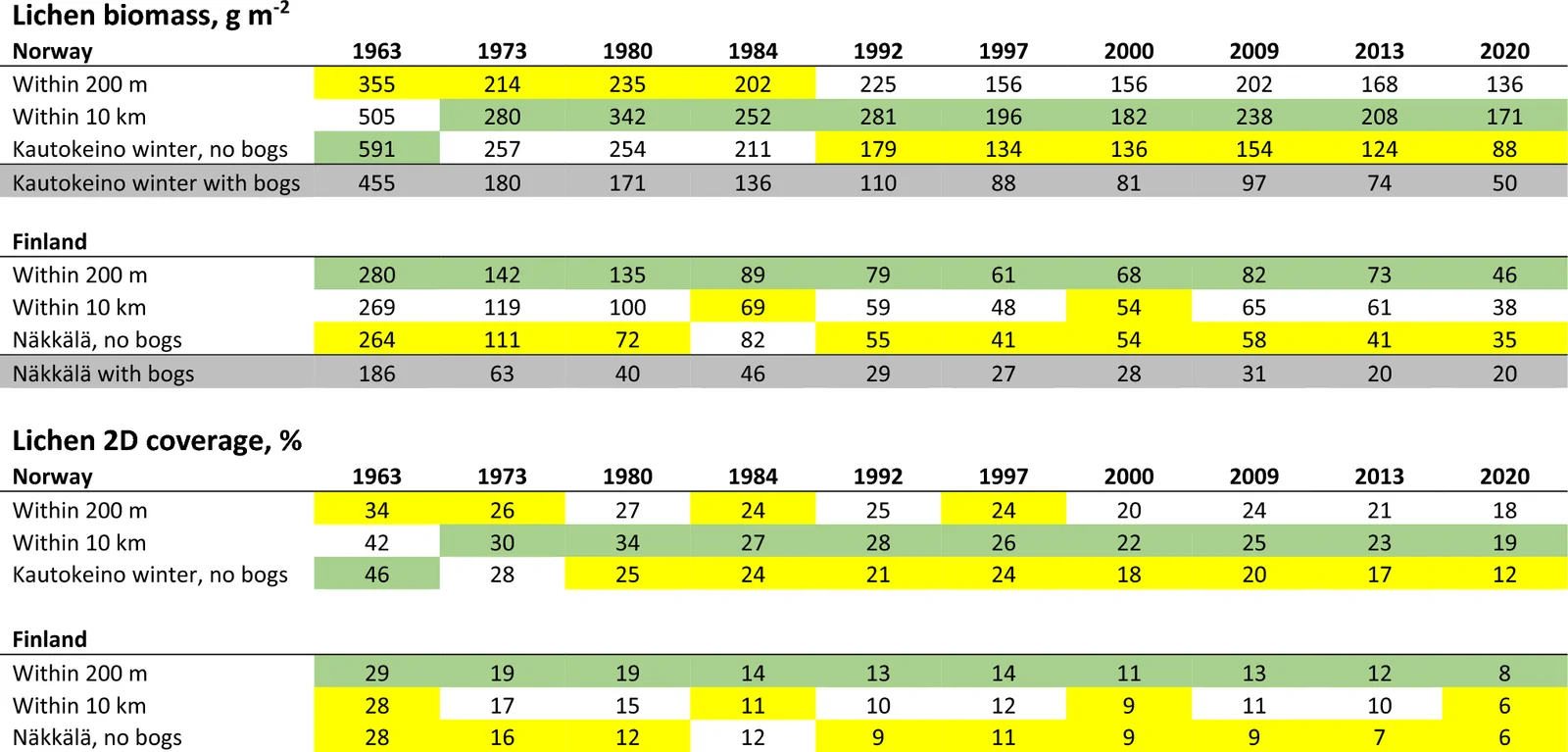
Comparison of the reconstructed forage lichen biomasses (g m^−2^) and 2D coverages (%) for different years and sections of the study area. Computations exclude forested areas and lakes. Peatlands are included in the herding district level with grey shading but excluded from the other calculations to make them comparable. Green shading shows the section with the best lichen pastures within the year, and yellow shading corresponds to the most worn-out section. When examining these results, it must be considered that the weighted mean absolute per cent error in comparison with independent field observations was as high as 35%, meaning that the true biomass in the landscape may have been considerably higher or lower in any of the individual time points

where ${O}_{i}$ is the lichen biomass based on independent field observations and ${S}_{i}$ is the biomass that we reconstructed using satellite images, and *n* is the number of temporal points. However, potential effects from differences in resolution between field studies and satellite images could not be taken into account.

### Modelling herding district lichen biomasses

To evaluate the causes behind lichen cover trends, we devised a model of forage lichen biomass on the herding district level. The model assumes that the reindeer population is the pivotal factor that affects lichen biomass. This is obviously a simplification of reality, as the model does not consider other external factors, for example icing events, that occasionally negatively affect the lichen cover (Bjerke 2011).

The model computes average forage lichen dry biomass *b*_*t*_ per square kilometre in the management area at the beginning of year *t* as follows:

$$b_{t} = b_{t - 1} + g_{t - 1} {-} \, e_{t - 1} {-} \, w_{t - 1} ,$$

where *b*_*t-1*_ is lichen biomass at the beginning of the previous year, *g*_*t-1*_ is lichen growth during the previous year, *e*_*t-1*_ is the lichen quantity eaten by reindeer during the previous year, and *w*_*t-1*_ is the quantity of lichen wasted by reindeer during the previous year (*t −* 1)*.* Wasted lichen refers to the lichen quantity that is removed from the biomass by reindeer but not eaten. A reindeer loses some food while eating, meaning that not all of the grazed lichen goes into digestion but instead falls onto the snow and ground. However, trampling is probably the most important cause of lichen wastage by reindeer (Heggenes et al. 2017). Annual lichen growth was estimated from data acquired from an ongoing long-term lichen growth experiment in Kautokeino and Karasjok in Norway (see Tømmervik et al. 2012). Annual relative lichen growth *R*_*t*_ in the plots (Fig. S5) was:

$$R_{t} = \, 1.95b_{t - 1}^{ - 0,1}$$

The highest annual lichen production (approximately 31 g m^−2^) was found in plots where the lichen biomass was ca. 300 g m^−2^. Lichen accumulation decreases, on average, to zero when biomass reaches 800 g m^−2^ on the plots (Fig. S6).

On the herding district level, lichen growth *g*_*t*_ during year *t* was estimated with the following equation:

$$g_{t} = f_{t} (b_{t - 1} R_{t} {-}b_{t - 1} ),$$

where *f*_*t*_ is a factor which defines lichen growth potential depending on the biomass distribution in the landscape. For example, if the average lichen biomass in a landscape is 100 g m^−2^, the plot-based data predict lichen growth of approximately 23 g m^−2^ (Fig. S6). However, the lichen biomass in the landscape can be distributed in such a way that that the lichen biomass grows clearly less than the predicted value. Given the example with 100 g m^−2^ average in the landscape, most of the area may have very small lichen biomass and therefore small absolute growth, while a fraction of the landscape has much higher biomass (e.g. 800 g m^−2^), which is not either markedly increasing because it is close to maximum. Factor *f*_*t*_ was estimated separately for the Näkkälä area and Kautokeino winter herding districts using a dense random point cloud to pick the biomass values from the reconstructed biomass maps. Factor *f*_*t*_ is approximated with the following equation:

$$f_{t} = \frac{{(\mathop \sum \nolimits_{r = 1}^{n} b_{tr} \left( {1.95 b_{tr}^{ - 0,1} ) - b_{tr} } \right)n^{ - 1} }}{{b_{t} (1.95 b_{t}^{ - 0,1} ) - b_{t} }},$$

where *b*_*tr*_ refers to the lichen biomass of random point *r* in year *t,* and *n* is the total number of random points. The *f*_*t*_ values varied in a narrow range (0.56–0.72), and the years that were not represented in the lichen biomass maps were interpolated from the closest values.

Lichen intake over 365 days or ‘one reindeer year’ (see explanation for reindeer numbers and densities in the next section) in the winter herding district was calculated, assuming that reindeer consumed only ground lichens to satisfy their energy requirements. The wintertime metabolic energy requirement of a middle-sized reindeer (a pregnant female with a body mass of 90 kg) is 20 megajoules (MJ) day^−1^ (Boertje 1985). The metabolic energy gained from 1 kg of dry matter of reindeer lichen is 10.8 MJ (Pekkarinen et al. 2015). Thus, the metabolized lichen biomass per reindeer year was estimated to be 676 kg (20 MJ/10.8 MJ kg^−1^ * 365 days) in the Kautokeino winter pasture and half of that (338 kg) in the pastures of Näkkälä on average. Hence, the ingested lichen quantity in metric tonnes km^−2^ (equal to g m^−2^) in year *t* follows the equation:

$$e_{t}\, = \, id_{t} ,$$

where *i* is the ingested quantity of dry lichen matter, the value being 0.676 for the winter pasture and 0.338 for the different pastures of Näkkälä on average. Parameter *d*_*t*_ is the average density of reindeer in the management area, calculated as reindeer year^−1^ km^−2^ (see the next section). The above formula probably overestimates the ingested quantity of ground lichens, at least in the winter pasture, considering that lichen rarely exceeds 80% in the reindeer diet (Kojola et al. 1995). However, we also run the model without any ingestion to quantify the effect of lichen ingestion on the landscape lichen biomass.

Lichen that is lost when reindeer forage was modelled as a function of the lichen biomass and reindeer density as follows:

$$w{t \, =\, } b_{t} d_{t} l,$$

where *l* is a factor for the loss of lichen biomass and depicts the annually upended proportion of the lichen biomass by one reindeer year^−1^ km^−2^. The value of *l* depends on the season and on the grazing regime.

#### Parametrizing the model with reindeer densities and loss factor

Annual average reindeer numbers in Näkkälä from 1946 to 2020 were acquired from the Reindeer Herders’ Association in Finland and from old volumes of the *Poromies* journal (Table S1). Data on reindeer numbers in Näkkälä for the years 1947–1953 were not found and therefore interpolated linearly from the 1946 and 1954 values. Reindeer numbers in the Kautokeino winter district were acquired from the Norwegian–Swedish Reindeer Herding Commission (1967) and the Directorate of Agriculture in Norway (Table S2). Reindeer are not allowed in the winter district between 1 June and 31 September, but the entire reindeer population only stays within the area for approximately 2.5 months in mid-winter (Käyhkö and Pellikka 1994). In practice, one reindeer year^−1^ km^−2^ in the winter pasture corresponds to a density of 2.3 animals km^−2^, with each individual spending 157 days there.

We used three biomass values (200, 400, and 600 g m^−2^) from 1946 to analyse model sensitivity. The values for loss parameter *l*_*s*_ were sought separately for the pastures of Näkkälä and for the Kautokeino winter district so that the model best-fitted with the reconstructed history of the lichen biomass in the herding areas. An Excel spreadsheet containing the reindeer data and the model is available online (Tables S1 and S2).

## Results

### Lichen cover reconstructions

Greyscale values of the drone images explained most of variation in the plot scale lichen 2D coverage (*r*^2^ = 78%) but less of the lichen biomass (*r*^2^ = 33%) in the Jauristunturit–Máðároaivi data used for fitting the regression model (Fig. 4a and b). The corresponding values for Palokorsa–Sieiddečearru site were 50% of the variation in lichen coverage and 30% in lichen biomass estimates at the plot level (Fig. 4). The data used for testing our equations included fewer plots with high lichen coverage and biomass than the training data, which may have caused somewhat poorer performance. The relationships of the greyscale values with the plot-level lichen cover were, however, highly significant (*p* < 0.001).

We got a clearly higher coefficient of determination at the landscape level (79%) when comparing our reconstructions with the values of the independent field surveys. However, the weighted mean absolute per cent error (wMAPE) was 35%, indicating considerable inaccuracy for district-level estimates (Table 3). Part of the error was due to the varying pixel size that resulted in 12–25% lower lichen biomass estimates for the coarsest resolution compared to the finest resolution (Table 4).

**Table 4:** High-resolution images resampled into coarser pixel sizes to test the effect of rescaling. A coefficient shows the proportion of the estimated forage lichen biomass for different resolutions compared to the biomass estimate from the original resolution

Image	Lichen biomass, g m^−2^	0.5 m	10 m	30 m	60 m
Drone image mosaic from Jauristunturit–Máðároaivi	227	1.00	0.91	0.89	0.86
Drone image mosaic from Palokorsa–Sieiddečearru	62	1.00	0.92	0.80	0.75
Sentinel-2, 2020	37		1.00	0.94	0.88

**Fig. 6:**
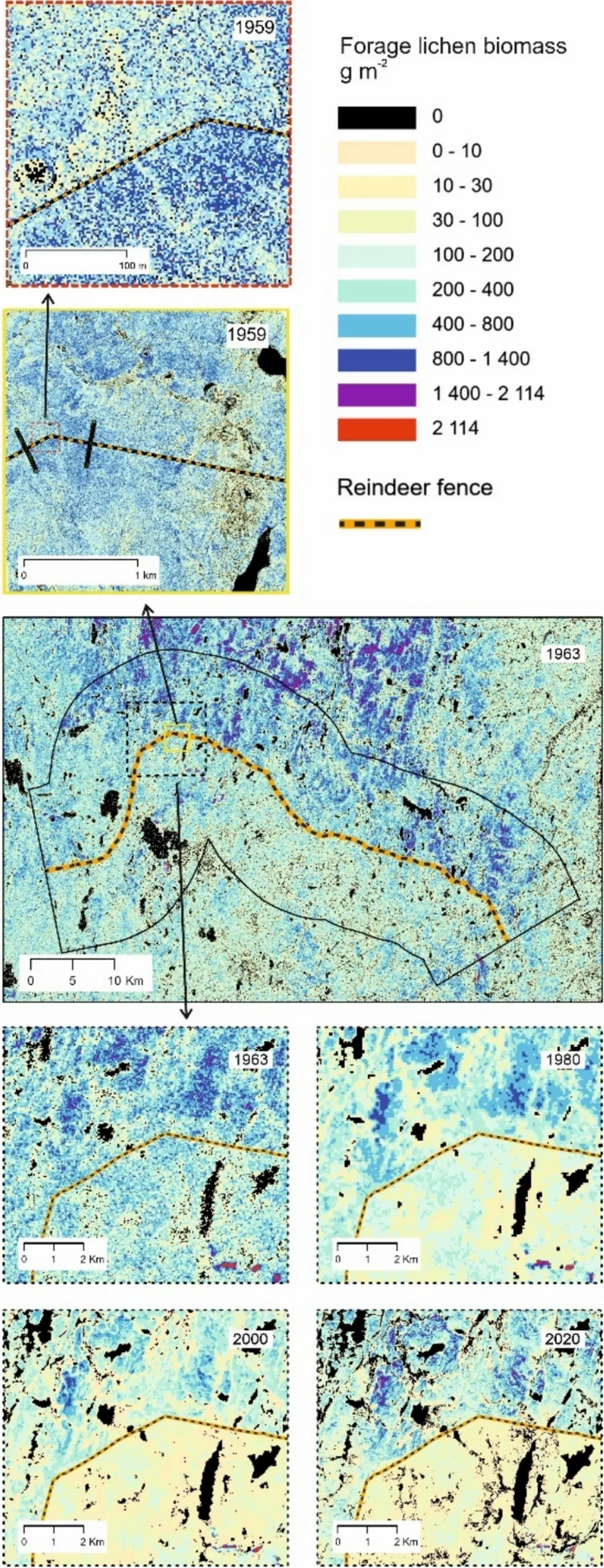
Lichen biomass maps for certain years and areas. Negative values were reclassified as zero, falling mostly into lakes and fens. Lichen maximum biomass was limited to 2,114 g m^−2^. That value is reached with the same raster value corresponding to 100% lichen 2D coverage. High reflectance values corresponding to the maximum lichen biomass and coverage can be produced by a very bright *Cladonia stellaris* mat but also by beaches and other sand surfaces formed through deflation

According to the 1959 aerial photographs, only a small difference existed in lichen cover between adjacent areas on the Finnish and Norwegian sides of the study area (Figs. 5, 6). The lichen cover ratio, i.e. the lichen cover in Norway divided by the lichen cover in Finland, was 1.04, indicating a slightly denser lichen cover on the Norwegian side. However, at some locations the lichen cover was even denser in Finland (Fig. 6). Unfortunately, due to limited areal coverage, the aerial images did not allow a comparison of the lichen cover between the entire herding districts.

Forage lichen cover markedly decreased on both sides of the fence from 1959 to 2020 (Fig. 6, Table 3). By 1963, the lichen biomass ratio was 1.9 within 10 km of the border fence, the 2D coverage being 28% on the Finnish and 42% on the Norwegian sides. In 2020, the lichen biomass ratio was 4.5, and the coverages were 6% and 19% in Finland and Norway, respectively.

The biomass reconstructions indicated that, the forage lichen biomass decreased on both sides of the border during 1963–1973 (Table 3). From 1973 to 1980, the decrease continued on the Finnish side but not on the Norwegian side. The decrease in lichen biomass continued on both sides of the border in the 1980s until 1997, after which the lichen pastures temporarily recovered until 2009 (Table 3). The direction of changes in the lichen cover has been mostly the same on both sides of the border. However, our results indicate opposite border fence effects in Norway and Finland. On the Norwegian side, the forage lichen biomass has been lower within the 200 m closest to the fence, compared to the area within 10 km from the fence (Table 3). The comparatively low lichen biomass next to the fence was especially clear between 1963 and 1980, but the phenomenon is still clear today in Kautokeino. In Näkkälä, the lichen biomass within the 200 m zone has been consistently higher than elsewhere in the herding district.

### The lichen biomass model

**Fig. 7:**
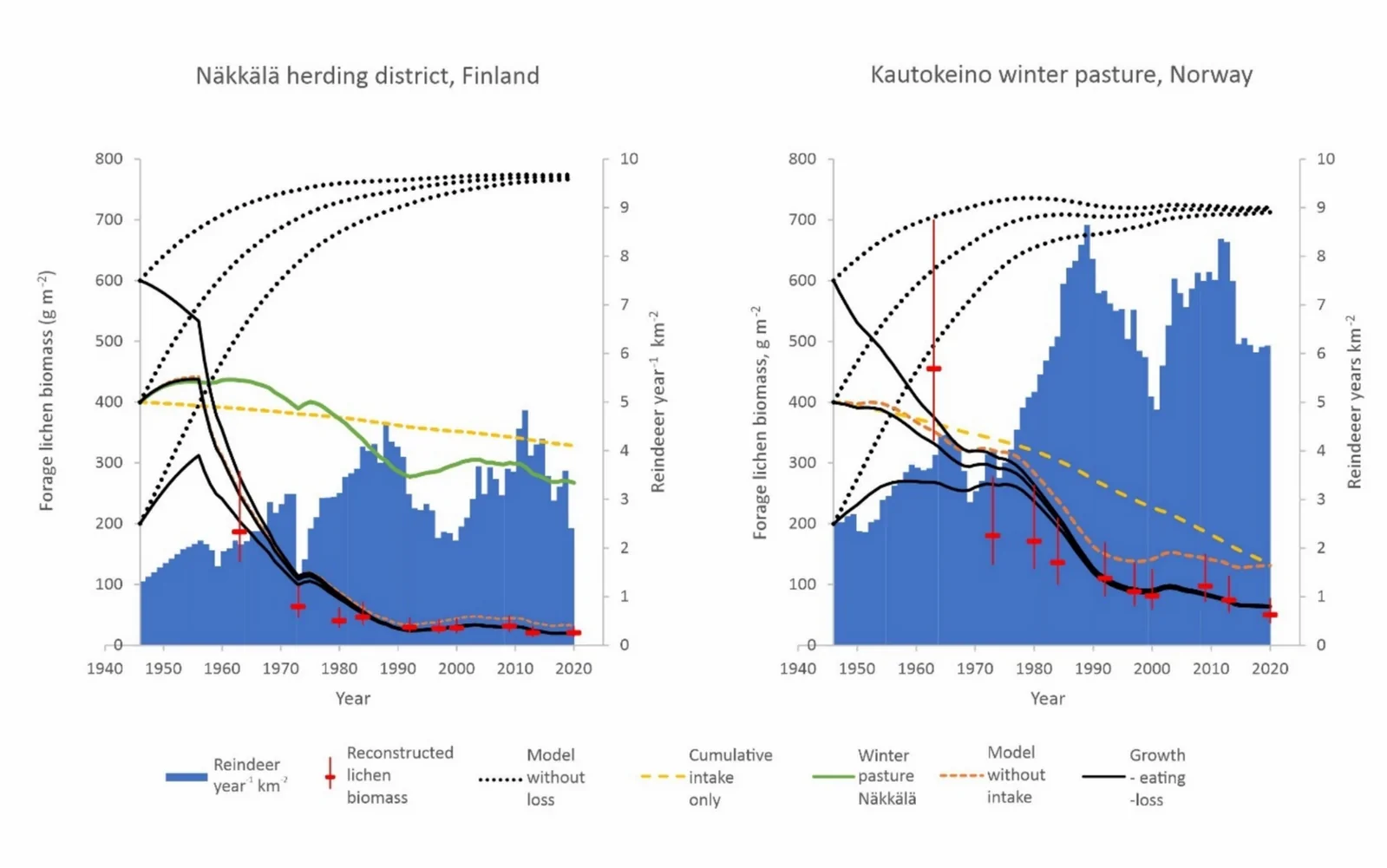
Herding district-level model for forage lichen biomass. The solid lines denote the full model runs from different initial values. The green line is a simulation for Näkkälä if it would be used only as a winter pasture, i.e. the loss factor would be equal to the Kautokeino winter district. “Cumulative intake only” refers to a model run without lichen growth and loss other than consumption. The dotted black lines show model runs without loss but with lichen growth and intake by reindeer included

In the landscape-level model for forage lichen biomass, loss parameter* l* was estimated to be 0.069 for the Näkkälä pastures and 0.019 for the Kautokeino winter pasture (Fig. 7). The results for the Kautokeino winter pasture during 1973–2020 indicate a loss of 1.4–5.1 times the annual intake (676 kg) by reindeer year^−1^, depending on the lichen biomass (Fig. 7; Tables S1 and S2). In Näkkälä, the corresponding loss was estimated to have varied between 4.1 and 12.9 times the annual intake (338 kg) by reindeer year^−1^.

Excluding all causes for lichen loss except consumption from the model resulted in a predicted lichen biomass increase to approximately 700 g m^−2^, despite the increase in reindeer population. Another model run without lichen growth and without other causes of loss except consumption resulted in gradually declining lichen biomass from the initially set value of 400 g m^−2^. A model excluding consumption but including loss and lichen growth produced close to similar results as the full model including consumption (Fig. 7).

According to our model, the quantity of lichen lost during grazing relative to the density of one reindeer year^−1^ km^−2^ depended on the lichen biomass in the area and on the pasturing type. For example, in the Kautokeino winter pasture in 1992, the model runs indicated forage lichen biomass being about 109 000 kg km^−2^ (Fig. 7), lichen growth of about 14 700 kg km^−2^, and a loss parameter of 0.019. This implies that approximately 2100 kg of biomass was lost per reindeer year^−1^ km^−2^. However, the total loss estimated for a density of 7.3 reindeer year^−1^ km^−2^ was 15 100 kg km^−2^, indicating that lichen biomass in the Kautokeino winter district was slowly declining. Correspondingly, the model suggests that in 1992, the Näkkälä pastures had, on average, 24 000 kg km^−2^ forage lichen biomass with a lichen growth of 6700 km^−2^ and a loss parameter of 0.069. This indicates a loss of about 1700 kg per reindeer year^−1^ km^−2^, the total loss being 5200 kg km^−2^, suggesting an increase in the lichen biomass for this year.

## Discussion

### Historical lichen cover decline

According to our first images, the 1959 and 1961 aerial photographs, the border zone in Finland and the Kautokeino winter pasture in Norway had relatively similar lichen cover in the late 1950s, which started to diverge at the beginning of the 1960s. The aerial photographs did not allow comparison of the whole herding districts. However, the reconstructions based on the satellite images indicate that in 1963, the Finnish Näkkälä reindeer herding district had clearly lower lichen biomass than the Kautokeino winter district, a difference which became more pronounced during the next two decades (Fig. 5, Table 3). Since 1980, the lichen biomass in the Kautokeino winter pasture has been 2.5–4.8 times higher than that in the Finnish Näkkälä pastures (Fig. 5, Table 3). However, a comparison of previously published lichen biomass values from Näkkälä (Mattila 1981; Kumpula et al. 2014, 2019) and Kautokeino (Tømmervik et al. 2009, 2012) herding districts suggests that lichen biomass is up to 13 times higher in Kautokeino than in Näkkälä. The large difference between studies is probably due to differences in focus area and analytical methods (see the methodological considerations below).

However, the higher lichen coverage and biomass on the Norwegian side must be evaluated, considering that the annual average reindeer density is generally twice as high (an actual wintertime density over four times higher) than on the Finnish side. The Finnish Näkkälä herding district sustains the reindeer population year-round, and winter and summer pastures are intermingled and partly overlapping (Stark et al. 2023). In contrast, the Norwegian side of the study area is only used as winter pasture. Thus, the higher lichen biomass in the Norwegian side, despite the higher reindeer density, could best be explained by the protective snow cover during the period when reindeer are present. However, since 1963, our reconstructed lichen biomass has declined by 80–90% also in Norway. Tømmervik et al. (2009, 2012) reported a similarly dramatic decrease for the Norwegian side of our study area, thus supporting our reconstructions.

Grazing is not the only factor that can affect lichen biomass. For example, He et al. (2024) reported a significant lichen cover decrease between 1980 and 2020 in Quebec, north-eastern Canada. They attributed 23% of the decrease to wildfires, while the cause remained unclear in 77% of the area, with shrub encroachment and caribou grazing listed as possible causes. In our study area, wildfires are rare and can be ruled out as the reason for the loss of lichen cover that occurred throughout the study area. Cornelissen et al. (2001) suggested that global change is promoting growth of vascular plants and the decline in lichen abundance. On the Norwegian side of our study area, Tømmervik et al. (2009) reported an increase in mountain birch forest cover and tree biomass in the region from 1957 to 2006. However, this cannot explain the observed decrease in lichen cover in the studied treeless tundra heaths (Fig. 5). In addition, increased nitrogen (N) deposition could also reduce lichen abundance (Gutiérrez-Larruga et al. 2020), but as N deposition levels are low in our study area, this probably did not make any marked contribution to the lichen decline. We suggest that the main causes for the decrease are direct and indirect effects related to the reindeer population and the altered grazing regimes, as discussed in the next section.

In our study area, the decrease in lichen cover in both herding districts coincided with the transition from nomadic migration to more modern reindeer herding and with the gradually increasing reindeer numbers (Table 1, Fig. 7). In Näkkälä, this transition of herding meant less active year-round herding after the completion of the border fence (Näkkäläjärvi 2007; Helle and Jaakkola 2008; Lehtola 2012). Especially less intensive summer herding may have caused the decline in lichen cover on the Finnish side as Näkkälä reindeer tend to ascent to the windy mountains next Norwegian border to escape mosquito season during June-July (Kitti et al. 2009). According to our lichen cover reconstructions, this part of Näkkälä has experienced the greatest loss of lichen cover (Fig. 6, Table 3).

Summer pasturage is not a valid explanation for the lichen decline in the Kautokeino winter district as the Norwegian reindeer migrate to the coast for mid-summer. Instead, we interpret this loss of lichen in the Kautokeino winter district as a result of two factors. First, the construction of the fence prevented the migration of reindeer. As indicated by the annual compensation requests between Norway and Finland (Anonymous 1954), part of the reindeer population continued their seasonal movements still in the 1950s. The Norwegian reindeer, trying to reach their old winter pastures deep within the Finnish side, were intercepted by the fence and may thereafter have caused detrition of the thick lichen cover on the Norwegian side. The 1959 aerial photographs clearly show sparser lichen cover in some places on the Norwegian side (see closeup for the year 1959 in Fig. 6.). This interpretation gets further support by the strong negative fence effect observed in 1963–1980. A similar fence effect on the Norwegian side was also observed by Tømmervik et al. (2012), as their study plots close to the border fence had lower lichen cover than the plots located further away from the fence (Tømmervik et al. 2012).

Despite some detrition of the lichen cover next to the border fence, our reconstruction from 1963 (in accordance with Lyftingsmo 1965) indicates that a majority of the lichen pastures within the Kautokeino winter district still had good lichen pastures in the beginning of the 1960s. The second important factor explaining lichen loss in the Kautokeino winter district may have been the introduction of snowmobiles in the 1960s (Pelto et al. 1968), which enabled more efficient use of previously underutilized remote parts of the Kautokeino herding district (Tømmervik et al. 2009). Given that lichen loss is proportionate to the standing biomass (Gaare and Skogland 1980; Pekkarinen et al. 2017), even a moderate reindeer number could significantly reduce a dense lichen cover.

The reason for the observed positive fence effect on the Finnish side, on the other hand, is unclear. It seems that reindeer avoid the proximity of the fence when they move in this border zone. Possible explanation is that the fence decreases wind speed and provides shelter for flying insects (Pasek 1988) which reindeer are trying to escape during summer.

### Potential impacts of trampling and other factors on lichen cover

On both sides of the border, the lichen biomass model adequately describes the reconstructed changes in lichen biomass in the landscape during the most recent decades (Fig. 7). However, the model required considerably higher lichen loss parameter for the Finnish Näkkälä district compared to the Kautokeino winter pasture. This difference had to be explained by something else than just consumption estimates as the model without loss other than intake indicates maximal lichen biomass for both sides of the border (Fig. 7).

The apparent reason for the differing loss parameters is that summertime trampling by reindeer occurs in the Finnish side but is absent in the Norwegian winter pasture. However, the model indicates that trampling and unspecified other forms of lichen loss *on both sides of the border* have been more important factors in explaining the decline than the sole intake by reindeer (Fig. 7). A major loss of lichen from trampling has generally been expected from summer pasturing but not winter pasturing (Tahvonen et al. 2014; Heggenes et al. 2017; Pekkarinen et al. 2017). However, Gaare and Skogland (1980) calculated that the wintertime loss was 2–10 times the quantity of lichen consumed by reindeer. This is in line with our results for the Kautokeino winter pasture during 1973–2020 indicating a loss of 1.4–5.1 times the intake (676 kg year^−1^), depending on the standing lichen biomass. The mechanism of lichen loss due to reindeer activity during winter time is not well understood. Nevertheless, there is evidence that small thallus fragments crumbled in snow by reindeer can provide a source of rapid lichen regrowth (Tømmervik et al. 2012).

The oldest reconstructed biomass values were in line with the oldest lichen biomass estimates from the Kautokeino winter pasture (Lyftingsmo 1965; Tømmervik et al. 2009, Table S3). Thus, there is a need to examine how the high biomass had accumulated in the first place. During World War II, the number of reindeer in our study area decreased by 30–35% (Alaruikka 1947; Tømmervik and Riseth 2011). Therefore, it is reasonable to assume that lichen biomass increased in the 1940s and early 1950s. On the Finnish side, prior to the completion of the fence, the model with an annual loss factor of 1.9% predicted the lichen biomass to be over 400 g m^−2^ with a population density of up to 1.7 reindeer years^−1^ km^−2^. However, on the Norwegian side, the reindeer densities were almost twice as high, and the same annual loss factor would have prevented the lichen biomass from accumulating higher than ca. 330 g m^−2^. This modelled value is lower than our reconstructions for 1963 and less than the oldest estimates by Lyftingsmo (1965) and Tømmervik et al. (2009), indicating that the loss factor was likely smaller than 1.9% before the 1960s. The reason for the assumed change in the loss factor on the Norwegian side is not clear, but it was coincidental with the transition from fully nomadic to semi-nomadic herding.

The higher lichen loss factor on the Finnish side could be explained by trampling during snow-free periods. Heggenes et al. (2017) estimated that reindeer may trample approximately 0.3 dm^3^ of lichen with each hoof print when walking on a 6–8-cm-thick lichen mat. During the summer and autumn, reindeer travel an average of 10 km a day (Reimers et al. 2014), resulting in approximately 40 000 hoof prints daily, thus potentially upending a volume of 10 m^3^ of lichen (Heggenes et al. 2017) whose dry weight is 220 kg according to our formula. This calculation pertains to a scenario where a reindeer continuously walks on a thick lichen mat, which is unlikely. Moreover, in humid conditions, the lichen mat largely recovers from trampling. However, considering the significant potential for trampling to damage the lichen cover, we suggest that reindeer trampling likely contributed to the decline, especially on the Finnish side of the study area. The trampling of lichen may have increased as herders reduced the continuous shepherding of reindeer during summertime after the fence was completed (Näkkäläjärvi 2007; Helle and Jaakkola 2008; Lehtola 2012; Table 1).

Besides the direct effects of reindeer, other factors may also have contributed to the changes in lichen cover (Bjerke et al. 2024; He et al. 2024). For example, climate change may have multiple impacts on lichens, including interactions with plants and herbivores. Different responses to warming by lichen, bryophytes, and vascular plants may act to decrease the niche space of lichens (Joly et al 2009). Changes in the density of other herbivores may interfere with the effects of reindeer on lichen abundance through plant community dynamics (Barbero-Palacios et al. 2024). Finally, our modelling approach necessarily relies on fixed terms for lichen growth and reindeer impacts, while the aforementioned factors and complex interactions may also have contributed to the observed decline in lichen biomass. Our results indicate a vast quantity of lichen lost to other causes than consumption, for which we believe the best explanation is trampling by reindeer. Further quantification of this effect should be sought from field experiments.

### Methodological considerations

The distinctively high reflectance of forage ground lichens proved a useful indicator that allowed us to produce plausible reconstructions of lichen cover at a landscape level. We detected a strong decreasing trend in lichen biomass on the Norwegian side of the border from 1963 to 2020, which aligns with previous long-term time-series analyses from the area (Tømmervik et al. 2009, 2012). We also detected a short-term increase in lichen biomass from 1997 to 2009, which could be attributed to lower reindeer densities following the harsh winters of 1996/1997 and 1999/2000. Those changes in lichen cover were previously known from field-based monitoring and remote sensing (Tømmervik et al. 2009, 2012). For lakes and for most open fens, our equations reliably predicted zero lichen cover and biomass. However, the computed weighted mean absolute per cent error was relatively high (35%), and the analysis relies on data collected from various remote sensing platforms with different sensors and resolutions; therefore, the details of our results should be interpreted with caution (Table 3).

Our biomass reconstructions tended to estimate higher lichen abundances than the estimates from previous vegetation inventories on the Finnish side, but lower estimates than inventories on the Norwegian side (Fig. 7). The discrepancy on the Finnish side is mostly due to the differences in lichen biomass calculations. In the equations used by Mattila (1981), one dm^3^ of dry *Cladonia stellaris* weighs 13.5 g, and one dm^3^ of *Cladonia rangiferina* is only 6.3 g. However, according to McMullin et al. (2011), dry 20 cm^2^ samples (which makes a volume less than one dm^3^ even if taken from a very thick lichen mat) of *Cladonia stellaris* weighs on average 46 g, *Cladonia rangiferina* 42 g, and *Cladonia arbuscula* 34 g. We used the value of 22 g dm^3^ for all forage lichens following the volume–weight estimate developed by Gaare and Tømmervik (2000) and Tømmervik et al. (2012) for sites where *Cladonia stellaris* cover was dominating (> 50%). In contrast, the nonlinear equation used by Kumpula et al. (2014) was based on a mixture of forage lichen species that were likely dominated by lighter species than *Cladonia stellaris*, and it yields at least one-third lower lichen biomass than our linear function for lichen coverage and height values typical for Finland. Additionally, the studied areas, habitats, and recorded species were not fully comparable among studies. For example, mires were included in our herding district-level calculations but not in the lichen inventories on the Norwegian side. Lichen cover and biomass are generally lower in mires than in tundra heaths within reindeer herding areas (Ahti and Oksanen 1990), but lichens are a characteristic feature of ungrazed northern bog hummocks (Jiroušek et al. 2022). However, most peatlands in our study area are wet Sphagnum mires lacking lichens (Kolari et al 2019).

Inaccuracies in the results can be expected when working with old and varying sources of imagery. For example, Landsat 1 and 2 satellite images from 1973 and 1980, which lack the blue channel, may underestimate lichen cover. This is because forage lichens, such as *Cladonia stellaris* and *Stereocaulon phascale,* reflect blue light relatively well compared to green plants like the dwarf birch (*Betula nana*) (Petzold and Goward 1988). However, a comparative analysis of the 1992 Landsat 5 images with and without the blue channel showed that excluding the blue channel did not have a large effect, as the blue channel added less than 4% to the estimated lichen biomass.

The varying pixel size also produced small errors in the study, as a coarser resolution resulted in smaller lichen biomass estimates (Table 4). A likely explanation is that a signal from small, isolated patches of lichens remains unnoticed in larger pixels. An analogous phenomenon has been reported in the land cover classification by Virtanen and Ek (2014); an increase in the pixel size of the source image reduced the estimated area of certain small and fragmented vegetation classes. Thus, the differences in resolution cannot explain the observed declining trend in lichen biomass. On the contrary, if all images had been of the same high spatial resolution, the trend would likely have turned out even steeper, as the old images with coarse resolution and lacking channels yield slight underestimates of the lichen biomass.

Starting from 1963, our reconstructions explained 79% of the variation in lichen biomass recorded in old field surveys (*p* < 0.001; Fig. 4e). This demonstrates the potential of our methodology in catching historical changes in lichen cover. With the prerequisite that suitable reference areas can be found, our mapping method can be utilized in similar landscapes when more sophisticated methods for lichen cover estimation (e.g. Kennedy et al. 2020; Erlandsson et al. 2022; Richardson et al. 2023) cannot be used due to limited spectral information in the old imagery. However, it must be kept in mind that our method includes several error sources that may vary depending on the area and the available remote sensing imagery.

## Conclusions

Lichen cover mapping based on greyscale imagery is possible due to the high reflectance of forage lichens. This method enabled us to quantify the historical decline in lichen biomass in contiguous reindeer herding areas in Finland and Norway, using variable imagery available over seven decades. However, it is not advisable to pick any individual time point of the reconstruction without considering the possible errors stemming from the cross-usage of multiple remote sensing data sources.

Differences in lichen biomass derived from remote sensing imagery across the fenced border between Finland and Norway can likely be attributed to differences in grazing patterns of reindeer herds in both sides. Our lichen biomass model indicates that direct intake of lichen biomass by reindeer appears to account for only a small proportion of the total lichen loss. Trampling during snow-free seasons, and interestingly, grazing related loss during wintertime seems to have a larger impact on lichen cover than intake by reindeer alone. We recommend controlled field experiments to quantify further the differences in lichen loss during summer and winter grazing and to increase the understanding of factors affecting lichen biomass.

While it may be challenging to restore abundant lichen cover with the current grazing regimes, it is notable that reindeer herding can be practiced even without very thick lichen mats, as demonstrated on the Finnish side of the study area, where reindeer herding has continued for decades, even with comparatively small ground lichen biomass.

## Supplementary Information

Below is the link to the electronic supplementary material.

## Data Availability

All satellite images used for analysis are freely available at https://www.onda-dias.eu/cms/ or at https://earthexplorer.usgs.gov/. Supplementary material containing the model for lichen biomass and data about historical reindeer densities are available on the journal's website as an Excel file.

## Footnotes

1 https://www.onda-dias.eu/cms/.

2 https://earthexplorer.usgs.gov/.
